# In situ electroporation of mammalian cells through SiO_2_ thin film capacitive microelectrodes

**DOI:** 10.1038/s41598-021-94620-8

**Published:** 2021-07-23

**Authors:** M. Maschietto, M. Dal Maschio, S. Girardi, S. Vassanelli

**Affiliations:** 1 Department of Biomedical Sciences, Section of Physiology, University of Padua, via F. Marzolo 3, 35131 Padua, Italy; 2 Padua Neuroscience Center, University of Padua, via Orus 2/B, 35131 Padua, Italy; 3 Institute of Condensed Matter Chemistry and Technologies for Energy, CNR, Corso Stati Uniti 4, 35127 Padua, Italy

**Keywords:** Biophysical methods, Gene delivery

## Abstract

Electroporation is a widely used non-viral technique for the delivery of molecules, including nucleic acids, into cells. Recently, electronic microsystems that miniaturize the electroporation machinery have been developed as a new tool for genetic manipulation of cells in vitro, by integrating metal microelectrodes in the culture substrate and enabling electroporation in-situ. We report that non-faradic SiO_2_ thin film-insulated microelectrodes can be used for reliable and spatially selective in-situ electroporation of mammalian cells. CHO-K1 and SH-SY5Y cell lines and primary neuronal cultures were electroporated by application of short and low amplitude voltage transients leading to cell electroporation by capacitive currents. We demonstrate reliable delivery of DNA plasmids and exogenous gene expression, accompanied by high spatial selectivity and cell viability, even with differentiated neurons. Finally, we show that SiO_2_ thin film-insulated microelectrodes support a double and serial transfection of the targeted cells.

## Introduction

A variety of methods are routinely employed to transfect mammalian cells, i.e. to introduce DNA molecules across the bi-lipid plasma membrane that physiologically separates the intracellular cytoplasm from the extracellular fluid. Among physical methods, electroporation is the most used^1^. Traditionally, it is performed in a cuvette on a population of cells in suspension between two facing metal electrodes (e.g. platinum). The field applied between the two electrodes has the purpose to cause the voltage across the cell membrane to change abruptly reaching a critical threshold (i.e., between 250 and 500 mV), thus leading to the opening of transient pores^2,3^ and providing a pathway to exogenous molecules to enter the cytoplasm^4–6^. In alternative, there is a growing interest for methods of *in-situ* electroporation, which avoid the potentially detrimental enzymatic and mechanical treatments to detach cells growing in adhesion on a solid substrate. In particular, recent developments include miniaturized and on-chip integrated microsystems that selectively operate in-situ electroporation on subpopulations of cells in adhesion^7–9^. With respect to bulk electroporation, these devices enable to operate spatially confined transfections of preselected subpopulations of cells^10,11^ or single cells^12,13^. In one approach cells are grown on top of microelectrode arrays integrated in the solid culture substrate. Selectivity is achieved because cells are targeted by stimulating the corresponding microelectrodes, which also enable serial transfection by repeating the electroporation procedure on the same target cells^13^. In an alternative configuration, the array of microelectrodes is approached from the top of the cell culture^14,15^. In general, in-situ electroporation allows for proper tuning of electroporation parameters, favouring transfection efficiency mainly thanks to the reduced intensity of the electric fields and their homogeneity across cells with respect to suspensions^16–19^. Moreover, microfabrication offers additional opportunities, e.g. to modulate the DNA concentration in the proximity of target cells by microfluidics^20^, by electrophoresis^21,22^ or by coating the surface with cationic polymers^23,24^. Noteworthy, with respect to electrodes design, 3D hollow nanoelectrodes were recently demonstrated to further reduce electroporation voltages^25,26^. In conclusion, on-chip microdevices based on integrated active sites are promising tools for selective in-situ electroporation and transfection of adherent cells, and has been proven with a variety of cell types including primary cultures^27–29^ and transfection molecules comprising DNA for exogenous gene expression or short oligonucleotides for RNA interference^27,30–33^. On the other hand, improvements are still necessary in terms of control over the field applied to the cells across a culture to optimize transfection reliability and efficiency and in terms of microelectrode stability for serial transfections or re-use. Metal-electrolyte interfaces, for instance, can suffer from non-linearities in the current–voltage relationship due to mixed faradaic and non-faradaic behaviour when subjected to large voltages (e.g., 1 V or above)^34^. This can be the case during field application in routinely used electroporation protocols. In this work, we demonstrate that in-situ electroporation of mammalian cells can be achieved efficiently through a SiO_2_ thin film that, similarly to other oxide films, is known to largely suppress faradaic currents^35–40^. We validate the method by demonstrating high transfection efficiency and cell selectivity for exogenous gene expression and by performing double serial transfections of adherent mammalian cells, including primary neurons.

## Results

### Capacitive electroporation of CHO-K1 and SH-SY5Y

**Figure 1:**
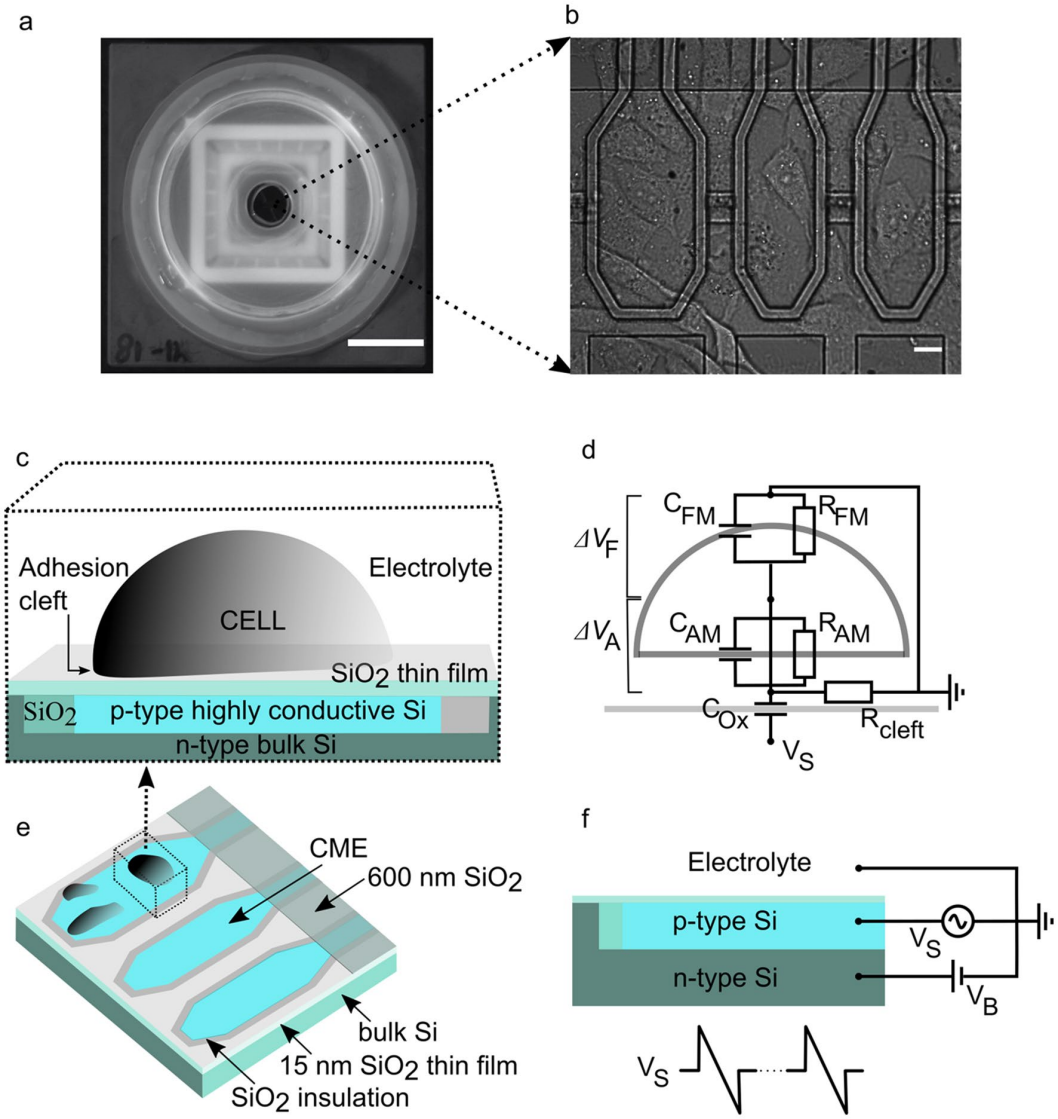
*Cells on SiO*_*2*_* thin-film capacitive microelectrodes*. (**a**) Top view of the electroporation microchip including the cell culture plastic chamber glued on a ceramic package providing mechanical support and electrical contacts to the voltage generator. Scale bar: 1 cm. (**b**) Magnification of three octagonal microelectrodes out of sixty-four (organized in two linear arrays as shown in Fig. 2) and with CHO-K1 cells growing in adhesion on top of them. Scale bar: 10 μm. (**c**, **e**) Drawing of the cell-chip interface and microelectrode structure (cross-section). The cell grows in adhesion on top of the SiO_2_ thin film from which it is separated by a narrow (i.e., in the range of a few tens of nanometers) cleft occupied by electrolyte. The highly conductive p-type silicon forms a capacitor together with the electrolyte and the oxide dielectric film separating the two. Insulation between microelectrodes is ensured by SiO_2_. (**d**) Equivalent electrical circuit of the cell-capacitive microelectrode (CME) system. The cell is described by two compartments of lipid membrane, one for the region of adhesion (with resistance R_AM_ and capacitance C_AM_), the other one for the free portion exposed to the bulk electrolyte (R_FM_ and C_FM_). R_cleft_ represents the resistance sealing the cleft from the bulk electrolyte. C_Ox_ is the capacitance of the oxide and V_S_ is the voltage applied to one face of the dielectric through the p-type Si. Accordingly, upon a change of V_s_ two potentials, Δ*V*_*F*_ and Δ*V*_*A*_, develop across the two compartments of the cell membrane, eventually causing electroporation. V_B_: is the bias voltage applied to the bulk n-type silicon. (**f**) Stimulation settings. The bath electrolyte is kept at ground potential through a Ag/AgCl electrode and the bulk n-type Si at a bias potential V_B_. A voltage source, V_S_, modulates the p-type Si. A typical stimulation waveform consisting of a repetition of sawtooth waves is sketched at the bottom.

The basic structure and operation of the electroporation chip with integrated an array of 32 × 2 SiO_2_ thin film capacitive microelectrodes is described in Fig. 1 (see also ‘The equivalent circuit’ in the Methods section)^41^. Each stimulation site consists of an octagonal microelectrode formed by highly conductive p-type silicon coated with a 15 nm thick layer of SiO_2_ (Fig. 1a–d). The electrolyte/SiO_2_/Si stack forms a capacitor, that is charged by applying voltage transients to the p-doped Si line realized on the substrate. The associated ionic current flowing into the cell-substrate cleft region causes a transient, non-null and localized extracellular field to which cells growing on top of the microelectrode are exposed (Fig. 1e–f). Each microelectrode is individually addressable.

**Figure 2:**
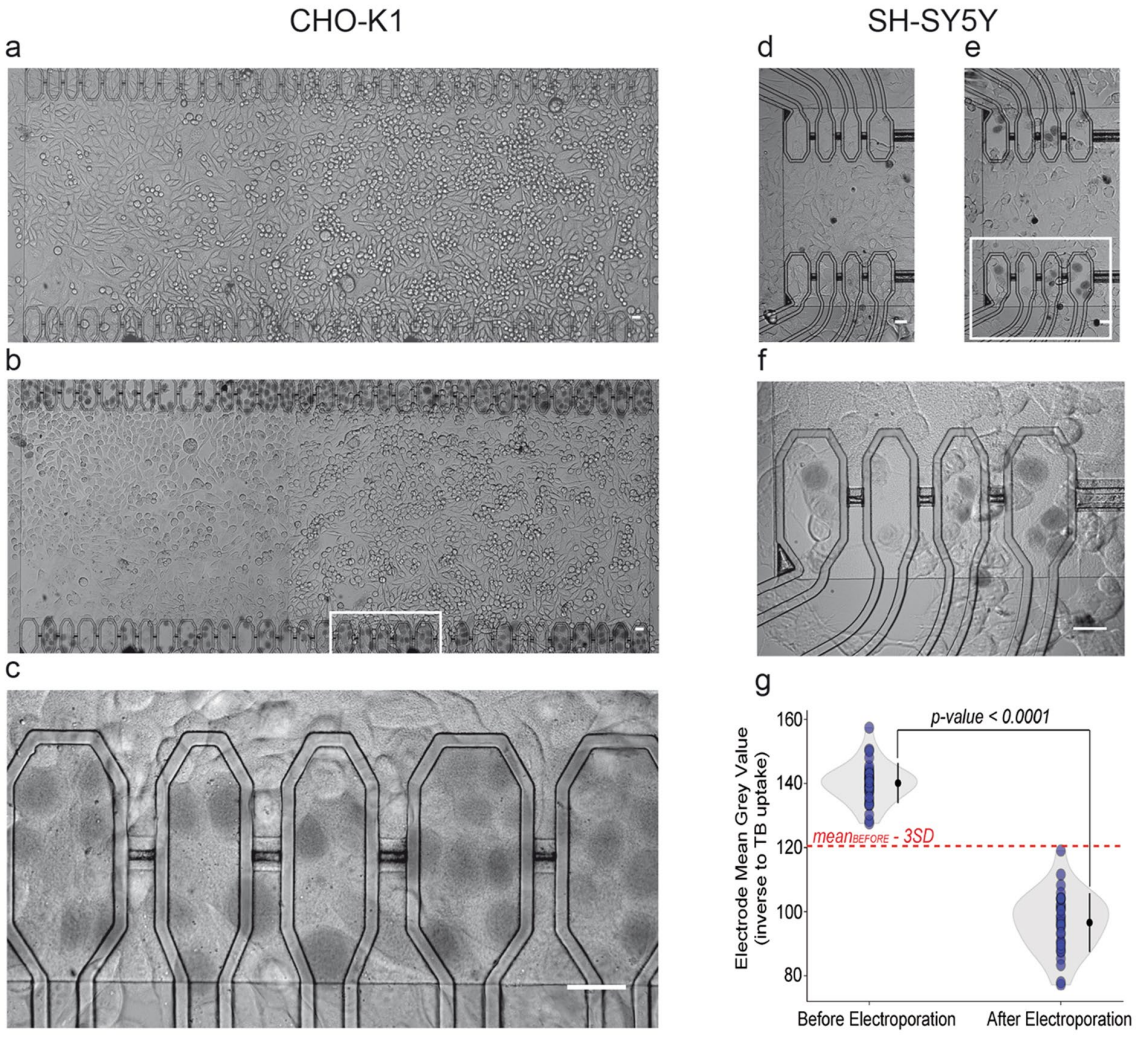
*Electroporation of epithelial CHO-K1 and neuronal SH-SY5Y cells*. (**a**) CHO-K1 cells growing on the SiO_2_ chip surface before electroporation. The two parallel arrays of capacitive microelectrodes covered by cells are visible at the top and at the bottom of the image. (**b**) Cells twenty minutes after electroporation with the buffer containing Trypan Blue (TB) and after rinsing. Cell morphology was preserved between *a* and *b*. (**c**) Magnification of five adjacent capacitors (white box in *b*) with TB stained cells. Scale bars (**a–c**): 20 μm. (**d**) SH-SY5Y cells on capacitive microelectrodes after one minute of pre-incubation with TB and rinsing. (**e**) Same cells twenty minutes after electroporation and washing. (**f**) Magnification of the white box in *e*. Scale bars (**d**–**f**): 20 μm. (**g**) Violin plot of grey levels of cells before and after electroporation with TB showing mean and SD (n = 32). The dashed red line indicates the threshold level of three SDs to determine the positive cells for electroporation.

We assessed the feasibility of capacitive electroporation in-situ by employing a high ionic strength electroporation buffer (see Methods) containing Trypan Blue (TB), an azo dye that does not permeate the cell membrane. CHO-K1 cells in culture on the SiO_2_ film were pre-incubated for one minute with the TB containing buffer, thus identifying cells with unspecific TB uptake and excluding them from subsequent analysis (Fig. 2a)^42^. Thus, all microelectrodes of the array were stimulated in parallel using the P1 voltage protocol (see Methods). After washing out, a clear pattern emerged: TB uptake was selective for cells in contact with capacitors and widespread across the array, thus demonstrating high efficiency (Fig. 2b, c). At the optical microscope, under visible light illumination, cells did not reveal significant morphological alterations hinting at a good recovery post-electroporation. Compared to CHO cells, neurons are known to be difficult to electroporate and to transfect^43^. Therefore, as a preliminary assessment, we tested capacitive electroporation on a neuroblastoma cell line, SH-SY5Y, using the same P1 protocol. The outcome in terms of TB uptake, selectivity and efficiency was similar to CHO cells (Fig. 2d–f).

We quantitatively analysed the TB uptake as a measure of electroporation by setting a threshold on a digital greyscale (i.e., from 0 for black to 255 for white) (see Methods). The violin plot shown in Fig. 2g reports the mean grey intensities calculated on 32 CHO-K1 cells before (140.108 ± 6.377; mean ± SD) and after (96.588 ± 9.326; mean ± SD) stimulation. Accordingly, cells were considered positive for electroporation and TB uptake when the grey intensity was below the mean computed before the stimulus by at least three standard deviations (dashed red line in Fig. 2g).

The overall electroporation efficiency was computed as the percentage of stimulated microelectrodes covered by at least one TB positive cell (see Methods). Noteworthy, we discarded from the count those cells that were showing TB uptake during incubation prior electroporation, and that therefore were deemed with a damaged membrane and possibly non-viable. The resulting efficiencies for CHO-K1 and SH-SY5Y cells were 81.46% ± 2.74% (n = 10) and 63.43% ± 3.00% (n = 5), respectively (mean ± SEM; n = number of electroporation experiments).

**Figure 3:**
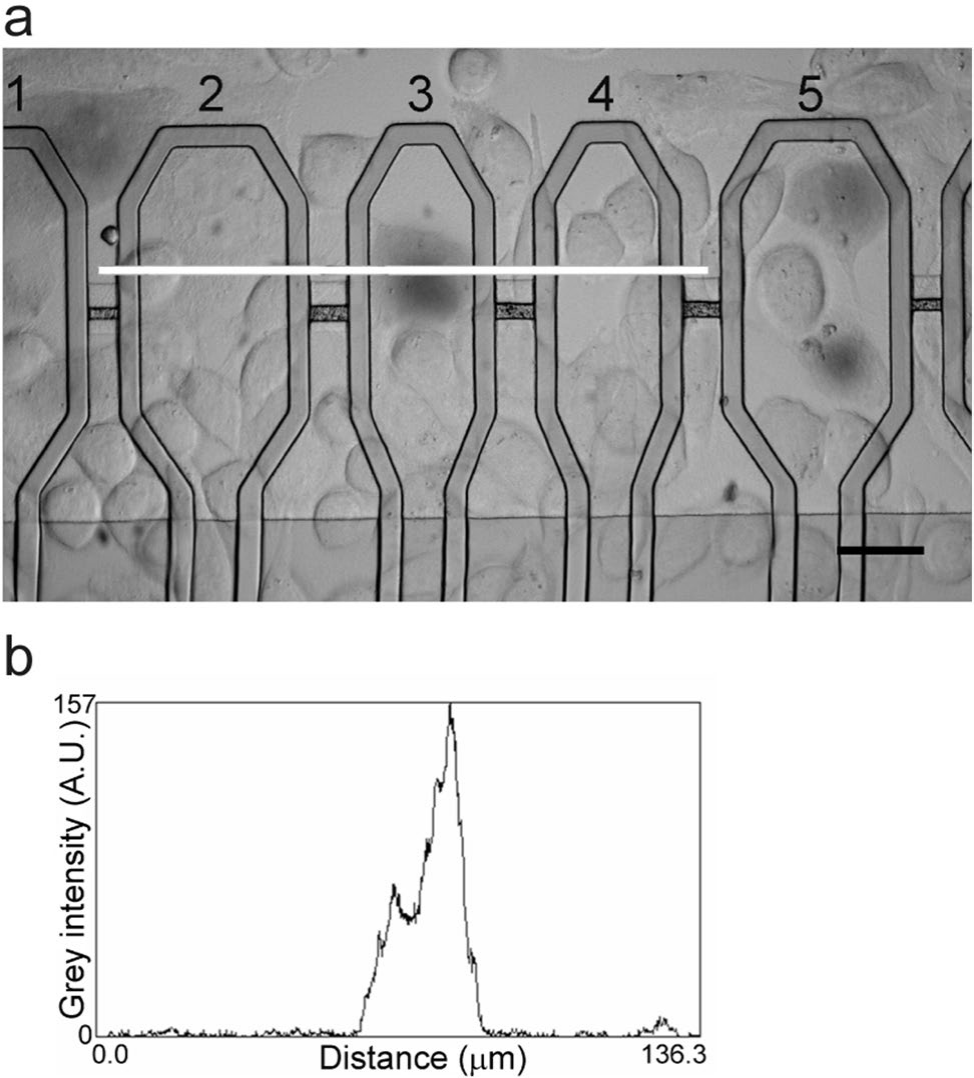
*Selective electroporation.* (**a**, **b**) Example of a selective electroporation assay with CHO-K1 cells. (**a**) Capacitors number 1, 3 and 5 were stimulated leading to selective TB staining of cells (scale bar: 20 μm). (**b**) Plot of grey intensity (expressed in arbitrary unit; A.U.) as measured along the white line in *a*. The intensity profile demonstrated the selective TB uptake by one cell on the stimulated microelectrode 3, and the absence of TB uptake on adjacent microelectrodes 2 and 4.

Selectivity of electroporation was further investigated by stimulating alternate capacitors. An example is shown in Fig. 3a (sites 1, 3 and 5 were stimulated leaving 2 and 4 silent). To exactly quantify TB uptake by cells, a grey intensity profile was measured along lines crossing capacitors. In the example of Fig. 3b, the selective electroporation-induced TB uptake by a cell on capacitor 3 is demonstrated in this way (Fig. 3b). Following this approach, we computed the selectivity index, *Sel*, for the whole array (see Methods) and demonstrated the high selectivity of the method both for CHO-K1 (0.88 ± 0.04, mean ± SEM; n = 7) and SH-SY5Y (0.88 ± 0.06, mean ± SEM; n = 6) cells.

### DNA transfection

**Figure 4:**
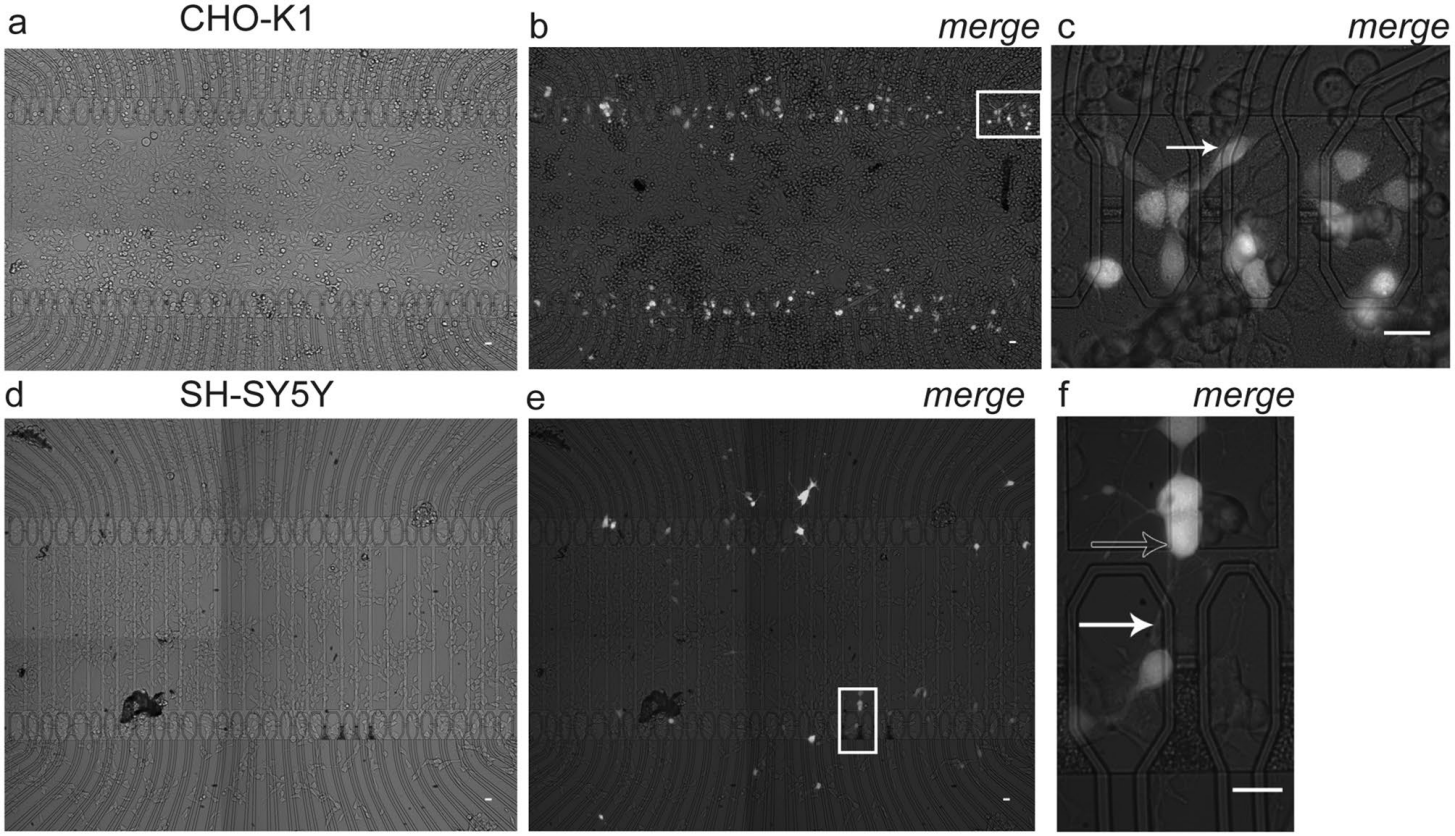
*Exogenous gene expression by capacitive electroporation*. (**a–c**) Transfection of CHO-K1 cells with a plasmid coding for EYFP. (**a**) Micrograph (visible light) of the cell culture 24 h after electroporation. (**b**) Merge of fluorescence and visible light microscopy pictures at 24 h post-electroporation. Fluorescent EYFP-expressing cells were found only on top of the capacitive microelectrodes. (**c**) Magnification of the white box in *b*. The white arrow points to a transfected cell. Scale bars: 20 μm. (**d**–**f**) SH-SY5Y transfection with EYFP. (**d**) Visible light micrograph of the culture 24 h after electroporation. (**e**) Merge of visible light and fluorescence microscopy images. (**f**) Magnification of the rectangular inset in *e*. One of the transfected cells (unfilled arrow) was covering the stimulated capacitor only with a branch (indicated by the filled white arrow), suggesting either that the plasmid was delivered into the process or that the cell migrated, perhaps following mitotic division, after transfection. Scale bars: 20 μm.

We developed an electroporation protocol, P2 (see Methods), with faster and short duration voltage transients for enhanced capacitive current injection to transfect CHO-K1 cells with a DNA plasmid. In these experiments we demonstrated the transfection using a plasmid coding for the Enhanced Yellow Fluorescent Protein (EYFP) (Fig. 4a–c). DNA in the electroporation buffer was at a concentration of about 30 ng/μl. Fluorescence microscopy observation at 24 h after transfection demonstrated a high transfection and gene expression efficiency (Fig. 4b). Most of the EYFP expressing cells were found on stimulation sites while a minority migrated in their close proximity (Fig. 4c). Overall, as for TB staining, the cell morphology was well preserved after electroporation, suggesting that the transfection protocol was preserving cell viability as clearly demonstrated by the later expression of the EYFP on most stimulated sites. EYFP expression was then performed on SH-SY5Y cells using the same protocol (Fig. 4d–f). Interestingly, cells could be successfully transfected also when branches or portions of the cell body were in contact with the capacitors (Fig. 4f). This was also observed for CHO-K1 cell bodies to a similar extent (Fig. 4c). In summary, the efficiency of EYFP expression (i.e., the percentage of microelectrodes with fluorescent cells over the number of stimulated microelectrodes) was 61.21% ± 5.87% for CHO-K1 (mean ± SEM; n = 9) and 23.08% ± 3.15% for SH-SY5Y (n = 10).

**Figure 5:**
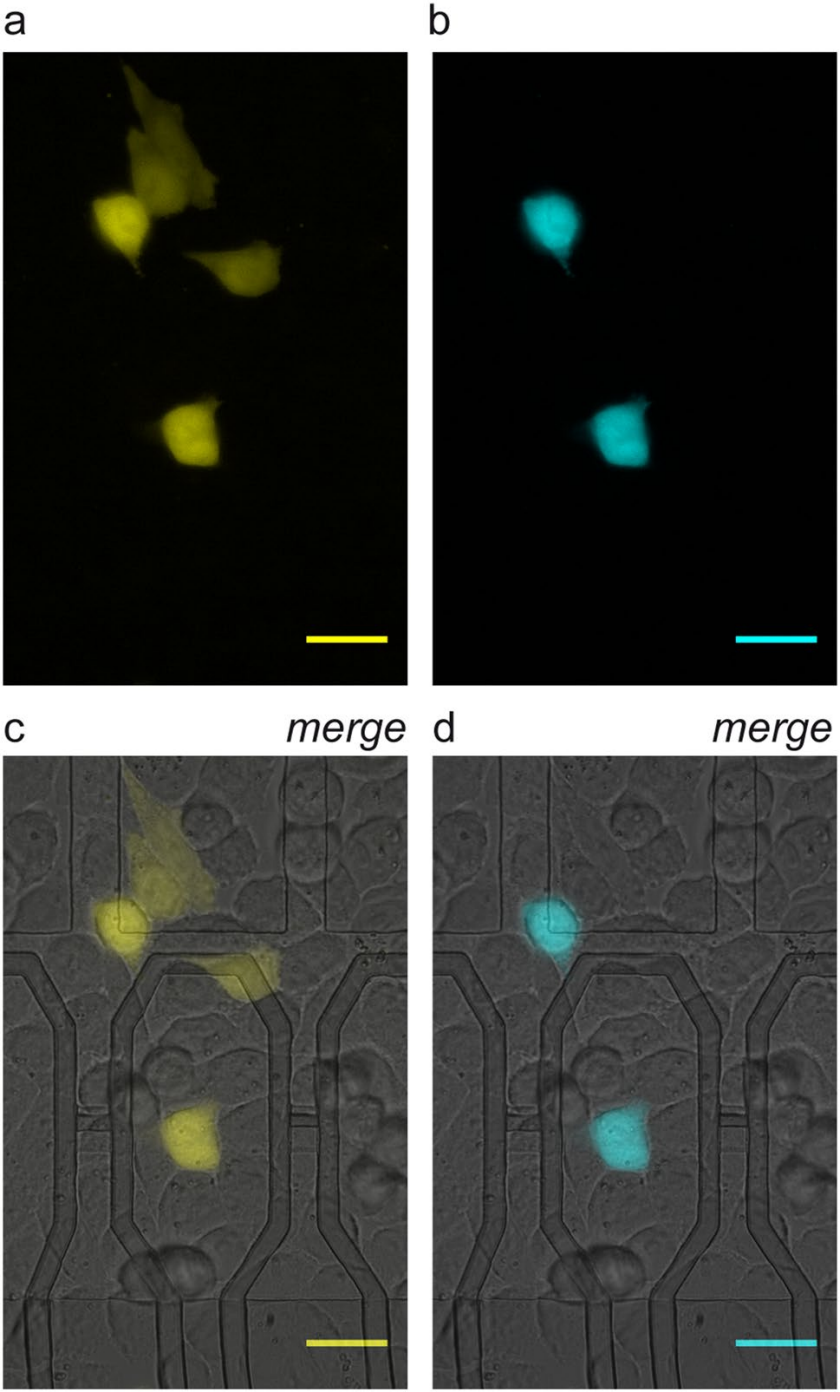
*Serial transfection with exogenous genes*. CHO-K1 were electroporated first with EYFP plasmid and 24 h later with ECFP. 48 h after the first electroporation, some of the EYFP expressing cells were also expressing ECFP: (**a**) EYFP channel; (**b**) ECFP channel. (**c**, **d**) Merged visible light and fluorescence micrographs. Scale bars: 20 μm.

Leveraging the results with in-situ capacitive transfection we set up a protocol of serial electroporation with DNA to obtain the sequential expression of two reporter genes. As shown in Fig. 5, after a first electroporation with DNA coding for EYFP, CHO-K1 cells were electroporated a second time 24 h later with a plasmid coding for the Enhanced Cyan Fluorescent Protein (ECFP). When observed at 48 h from the first transfection with EYFP, a subpopulation of the cells co-expressed ECFP (Fig. 5b, d) and EYFP (Fig. 5a, c). Noteworthy, although many transfected cells remained on microelectrodes, part of them migrated during the incubation period between transfection and gene expression observation and were found in their proximity.

### Transfection of primary neurons in culture

**Figure 6:**
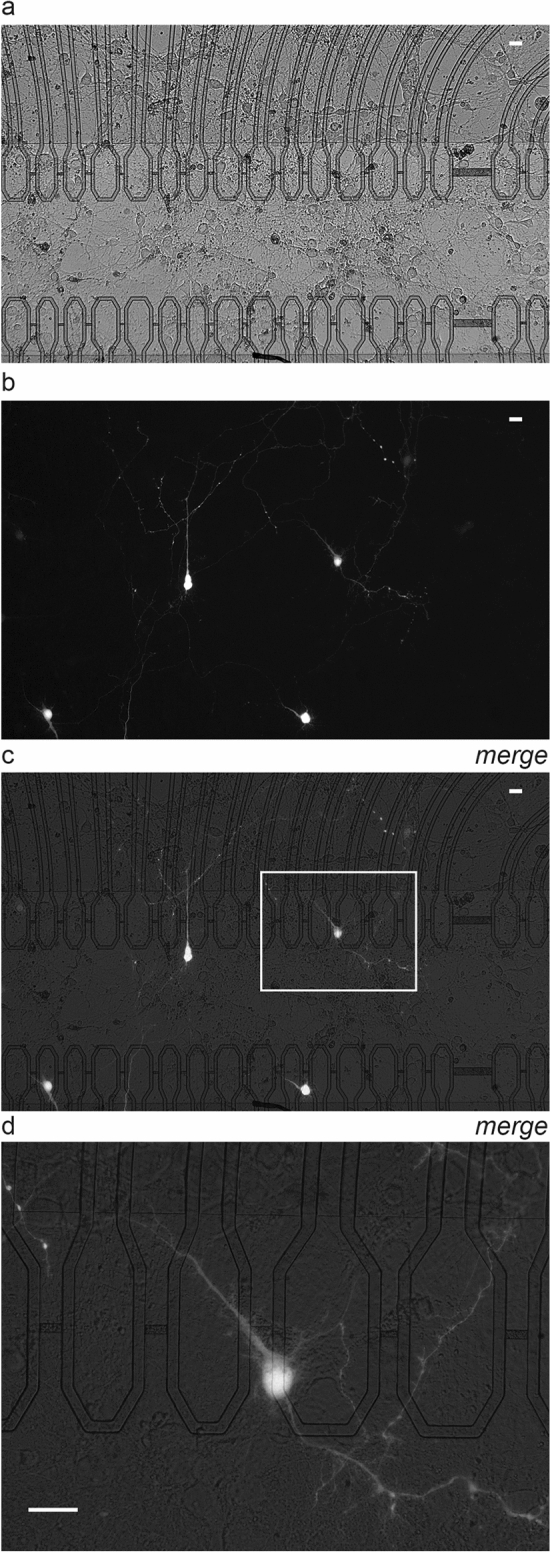
*In-situ transfection of primary neurons*. EYFP expression in E18 rat hippocampal primary neurons in culture transfected at DIV6 and observed at DIV7: (**a**) visible light, (**b**) fluorescence, (**c**) merged images. (**d**) Magnification of one transfected neuron (white rectangle in *c*). Scale bars: 20 μm.

After the mammalian cell lines, we assessed capacitive transfection on primary cultures of dissociated embryonic rat hippocampal neurons. Cells were electroporated at 6 days in culture with DNA coding for EYFP. Twenty-four hours after electroporation, a subset of the targeted neurons displayed the fluorescent signal associated with the transfected gene (Fig. 6a, b, c). As previously observed with SH-SY5Y, electroporation of branches was sufficient, in some cases, to deliver the plasmid to expression of EYFP in the whole neuron (Fig. 6d). As expected from the known difficulty to electroporate primary neurons, the expression efficiency was lower than for cell lines and generally below ten percent.

## Discussion

We have demonstrated the possibility to use thin film capacitors for electroporating mammalian cells grown in adhesion. By driving the capacitors with properly applied voltages, the plasmatic membrane of cells of different origin, ranging from the epithelial cell line CHO-K1 to primary neuronal cells, was transiently electroporated allowing the delivery of the TB vital dye and DNA plasmids. A single sawtooth wave of 0.5 s duration (i.e., 2 Hz frequency) was sufficient for the delivery of the impermeable vital stain Trypan Blue with high efficiency both in CHO-K1 and SH-SY5Y cell lines. By increasing the frequency to 10 kHz and the number of cycles to ten thousand, cells could be transfected with DNA plasmids encoding for fluorescent proteins. Noteworthy, as reported in the Methods, the frequency increase was accompanied by a significant increase of the slope of the sawtooth, thus generating an electric field of higher amplitude in the electrolyte through the capacitor. It is plausible that, under these conditions, exposed cells were developing longer lived and larger pores enabling DNA entry. The efficiency of DNA electroporation varied from about 60% for CHO-K1 to 20% for SH-SY5Y. These outcomes are comparable with previous results using lipidic transfecting agents for the delivery of a non-viral expression vector, where the high efficiency of CHO-K1 transfection (60–80%) was drastically reduced to 5% for SH-SY5Y cells^44^. Our experiments, besides demonstrating an improved efficiency for SH-SY5Y cells, paved the way for the use of in-situ capacitive electroporation for serial gene transfection of cells in adhesion. By transfecting CHO-K1 cells two times in sequence with EYFP and ECFP coding plasmids, we demonstrated the possibility to perform time-resolved electroporation for delivery of two compounds in series. This implied that cells recovered completely after both the first and the second electroporation. Given the well-preserved viability of cells after electroporation, we foresee that the approach could be exploited in the future for delivery of molecules at different times during the cell life span for time-resolved genetic manipulation.

In general, in-situ capacitive electroporation of cells in adhesion may offer a better yield in terms of genetic manipulation with respect to other approaches. In standard electroporation in cuvette, bulk cell cultures are exposed to an electrical field that is not homogenous across the cell population. In turn, this negatively affects viability. On the other hand, the use of chemical agents for transfection may compromise DNA expression in the cell. Recently, in mouse cell cultures transfected by lipofection with commercial reagents, a widespread gene expression modification was detected that was negatively affecting both heat-inducible stress genes and other genes involved in vital cellular functions (like metabolism, cell cycle and apoptosis^45^). With capacitive in-situ electroporation, adhesion to microelectrodes and minimization of faradic currents ensure more homogenous conditions of exposure to the electric field across cells, thus enabling a better refining of the electroporation process.

Contrary to cell lines, embryonic neuronal cultures are notably challenging to transfect when already differentiated, so limiting the possibilities for studies at later differentiation stages. EYFP coding plasmid transfection with efficiencies around 20 to 30% has been obtained in murine neurons electroporated in cuvette only immediately after dissociation from the hippocampi and before plating^46^. In this paper we have shown DNA transfection of rat hippocampal neurons at six days after plating, i.e. at a stage when the differentiation of neurons and neurite extension is almost complete^47^. Noteworthy, electroporation and the delivery of the plasmid inside the neurons was also obtained by stimulating a sub-region of the dendritic tree that was covering the capacitor area. On the other hand, the efficiency of electroporation of neurons remained low and below ten percent. However, according to the available literature, also nucleofection that enable plasmids to directly enter the nucleus, provides good efficiencies only with freshly isolated cells that have not yet developed branches^48^. Our approach appears to partially overcome this limitation and represent a complementary transfection strategy to target mature neurons in culture.

## Methods

### Microchips

A total number of 20 microchips were used for cell culture and electroporation experiments. Each chip contained two linear arrays of octagonal capacitive microelectrodes with a 15 nm SiO_2_ film and whose structure and manufacturing process are described in detail elsewhere^41^ (see Results and Fig. 1). A plastic chamber was mounted on the chip for cell culture and stimuli delivered to the capacitive microelectrodes through a function generator (33250A 80 MHz, Agilent Technologies). Chips could be re-used multiple times (approximately 60, in average) before incurring in deterioration or rupture of capacitive microelectrodes. The integrity of the oxide film was monitored periodically throughout the experiments by DC I-V measurements and by assessing the response to pulsed waveforms with a capillary Ag/AgCl microelectrode immersed in the electrolyte and measuring the potential above the stimulation site.

Briefly n-type silicon wafers (diameter 100 mm) with a (100) surface and a resistance of 0.45 ± 0.75 Ω cm were used^41^. Local oxidation of silicon (LOCOS) was employed to insulate the stimulator areas with SiO_2_. Highly conductive leads to the stimulation sites were created by bulk Si implantation with boron. A 660 nm passivation oxide was deposited by plasma enhanced chemical vapor deposition (PECVD) all over the chip and removed in the central area by etching. The 15 nm film of SiO_2_ was grown by dry thermal oxidation (TCA/O_2_ ambient) at 900 °C. The relative permittivity of the oxide estimated in electrolyte by impedance spectroscopy was about 4 (Birkenmeier & Fromherz, personal communication). The octagonal shape of the microelectrodes was chosen to reduce sharp edge effects on the electric field in the electrolyte.

Before each use, the microchips were gently cleaned with 5% (v/v) 70 °C Tickopur R33 detergent (Bandelin) for 1 min, sterilized for at least 30 min with 70% (v/v) ethanol, rinsed with sterile deionized water and air-dried under a sterile laminar hood. Thus, after addition of the cells suspension and seeding, they were kept in the cell incubator.

### The equivalent circuit

In general, the physical system of a cell in adhesion to a substrate takes into account the electrical properties of the cell membrane, represented by the membrane capacitance and resistance, and the physical properties of the cell-substrate junction, represented by the resistance (R_cleft_) to the charge flow along the electrolytic conductor of the cleft region toward the bulk electrolyte (Fig. 1). The Electrolyte Oxide Silicon structures serving as stimulation sites are represented as a series of a voltage source (V_S_) and a capacitance due to the oxide thin layer (C_Ox_). The low (28 Ω) series resistance offered by the conductive lane to the capacitive microelectrode is omitted from the drawing of Fig. 1. V_B_ is the bias voltage applied to the bulk n-type silicon. The cell is described by two compartments, one for the portion of the membrane in the cleft (R_AM_, C_AM_) and one for the membrane outside the adhesion area (R_FM_, C_FM_)^35^.

### Cells

The cell lines CHO-K1 (epithelial cells from Hamster ovary; kind gift by Dr. M. Zaccolo, Department of Physiology, Anatomy and Genetics, University of Oxford) and SH-SY5Y (from human neuroblastoma; kind gift by Dr. S. Guarnieri, Dipartimento di Scienze Mediche di Base ed Applicate, Università di Chieti-Pescara, Italy) were maintained in F-12 Nutrient Mixture—Ham—and DMEM media, respectively, both supplemented with 10% (v/v) heat-inactivated FBS (Foetal Bovine Serum), 10 u/ml penicillin and 10 μg/ml streptomycin, in a humidified atmosphere at constant temperature (37 °C) and CO_2_ concentration (5% v/v). Two days before the electroporations, 2 × 10^4^ and 10^4^ cells/cm^2^ were plated on chips for experiments with Trypan Blue and plasmids, respectively.

Wistar rats (Charles River) were maintained in the Animal Research Facility of the Department of Biomedical Sciences (University of Padua) under standard environmental conditions. All the procedures involving animals were realized according to Italian regulations for animal welfare (ethics approval from the Italian Ministry of Health, authorization number 522/2018-PR) and in compliance with the ARRIVE guidelines. Primary neurons were dissociated with papain from freshly dissected E18 rat embryos hippocampi as described previously^49^. About 3 × 10^4^ neurons/cm^2^ were plated on chips (pre-coated with 10 μg/ml poly-L-lysine; Sigma-Aldrich), maintained in NeuroBasal™ Medium supplemented with 2% B-27 and transfected at DIV6.

Culture media and transfection reagents, if not otherwise indicated, were purchased from Gibco™ (ThermoFisher Scientific).

### Electroporation

Stimuli were delivered through the function generator and monitored by an oscilloscope (54641A 350 MHz, Agilent Technologies). A standard Ag/AgCl electrode was immersed in the electroporation solution as ground reference. Two different electroporation protocols based on repetitions of sawtooth waveforms were used:

$${\text{P}}1:{\text{Slope}}\;\Delta V/\Delta t = - \,12\,{\text{V}}/{\text{s}},\;{\text{Frequency}} = 2\;{\text{Hz}};\;{\text{Amplitude}} = \pm \,3\;{\text{V}};1\;{\text{cycle}}$$

$${\text{P}}2:{\text{Slope}}\;\Delta V/\Delta t = - \,6 \times 10^{4} \,{\text{V}}/{\text{s}},\;{\text{Frequency}} = 10\;{\text{kHz}};\;{\text{Amplitude}} = \pm \,3\;{\text{V}};10,000\;{\text{cycle}}$$

A bias voltage V_B_ = 0 V was applied to the n-type Si.

In both protocols the maximum voltage amplitude range applied was ± 3 V, and well within the limits of oxide breakdown. All the electroporations were carried out in a small volume (70 μl) of a home-made buffer (named B1; in mM: 4 KCl, 10 MgCl_2_, 82.2 Na_2_HPO_4_, 37.8 NaH_2_PO_4_; pH 7.4; ionic strength: 339.8 mM; conductivity: 16,211 ± 252 μS/cm, n = 3). Prior to electroporation, the culture medium was removed, and the cells rinsed with PBS (in mM: 137 NaCl, 2.7 KCl, 10 Na_2_HPO_4_, 2 KH_2_PO_4_). Transfected cells were monitored in PBS at room temperature with the fluorescence microscope Olympus BX51WI (Olympus), equipped with 10X (numerical aperture 0.30) and 40X (numerical aperture 0.80) objectives, a CCD DFC350FX 12 bit grey scale digital camera (Leica) for images acquisition, and the following filters: for ECFP, excitation BP430/20, emission D480/40, dichroic DM455; for EYFP, excitation BP 477/35, emission HQ535/40, dichroic DM505. Pictures were acquired with LAS V4.5 (Leica Application Suite) software (Leica) and then digitally modified to add scale bars, pseudo-colors and useful line-arts (lines, arrows or boxes) with the open source ImageJ 1.49 s software (https://imagej.nih.gov/ij/).

### Transfected molecules

The electroporation was realized with two kinds of molecules, solubilized in B1 buffer:

- Trypan Blue (TB, Sigma-Aldrich; 0.08% in 70 μl of B1), a dark blue vital stain that enters a cell only if the plasmatic membrane is porated or damaged. It was used when applying protocol P1, to verify the reliability and selectivity of electroporation with capacitive microelectrodes. Before applying the stimulus, TB was incubated 1 min on the cell culture to detect any damaged cell. Twenty minutes after the electroporation, the TB solution was removed, the cells rinsed thrice with PBS and the result checked at the microscope;
- pcDNA3.1(-)EYFP/ECFP (2 μg in in 70 μl of B1), with cloned Enhanced Yellow Fluorescent Protein (EYFP) or Enhanced Cyan Fluorescent Protein (ECFP) gene^49^. Plasmids were delivered to the cells by means of protocol P2. After the electroporation, the plasmid solution was incubated twenty minutes on the cells, then the culture was rinsed thrice with PBS and put in the incubator with the appropriate sterile complete medium. The expression of the fluorescent protein EYFP or ECFP was verified 24 or 48 h after the electroporation.

### Quantification of TB uptake

To discriminate between TB uptake before and after electroporation, a threshold level was set basing on the cellular grey levels in images acquired after the incubation with TB (before electroporation). For a given chip, the mean grey levels in round regions of interest (ROI) with 105.843 μm^2^ area was calculated with the ImageJ 1.49 s software in 32 cells on capacitors (e.g., one randomly selected cell for each capacitor of the top linear array in Fig. 2a, b). The dimension of the ROI was chosen to fit to the body of cells of different morphologies. Mean grey values and standard deviations were calculated for the two experimental conditions “before electroporation” (that is, after 1 min of incubation with TB) and “after electroporation”, and the corresponding violin plot was created. TB uptake in a cell after electroporation was considered positive if the mean grey level value was less than mean grey_before electroporation_ – 3 × SD_before electroporation_.

### Efficiency and selectivity analysis

For each experiment the efficiency of electroporation has been calculated as Eff = (PS/S)*100, where “PS” represents the number of capacitors carrying at least one electroporated cell, “S” is the number of capacitors covered by cells and used for electroporation. Similarly, it was computed the transfection efficiency counting the capacitors with fluorescent cells.

To estimate selectivity, capacitors with cells were stimulated in presence of TB and the selectivity was determined by computing the index Sel = (PS − PNS)/PS, where “PNS” is the number of non-selected capacitive microelectrodes (i.e., with nonspecific positive cells). Thus, the index ranges between 0 and 1 with 1 indicating maximum selectivity. Noteworthy, all the selectivity experiments were performed after 1 min of pre-incubation with TB and excluding from the procedure those capacitors with cells displaying a spontaneous pre-electroporation uptake of the marker.

### Statistical analysis

Statistical analysis was done with Graph Pad Prism (Graph Pad Software). Comparisons between data groups were expressed as mean ± SEM.

## Data Availability

The datasets generated and/or analysed during the current study are available from the corresponding author SV on reasonable request.
